# The genetic background of acromegaly

**DOI:** 10.1007/s11102-017-0789-7

**Published:** 2017-02-04

**Authors:** Mônica R. Gadelha, Leandro Kasuki, Márta Korbonits

**Affiliations:** 1 Neuroendocrinology Research Center/Endocrine Section and Medical School - Hospital Universitário Clementino Fraga Filho, Universidade Federal do Rio de Janeiro, Rio de Janeiro, Brazil; 2 Neuroendocrine Section - Instituto Estadual do Cérebro Paulo Niemeyer, Secretaria Estadual de Saúde do Rio de Janeiro, Rio de Janeiro, Brazil; 3 Endocrine Unit, Hospital Federal de Bonsucesso, Rio de Janeiro, Brazil; 4 Centre for Endocrinology, Barts and the London School of Medicine, Queen Mary University of London, Charterhouse Square, London, EC1A 6BQ UK

**Keywords:** Acromegaly, Genetic basis, Familial disease, AIP

## Abstract

Acromegaly is caused by a somatotropinoma in the vast majority of the cases. These are monoclonal tumors that can occur sporadically or rarely in a familial setting. In the last few years, novel familial syndromes have been described and recent studies explored the landscape of somatic mutations in sporadic somatotropinomas. This short review concentrates on the current knowledge of the genetic basis of both familial and sporadic acromegaly.

## Introduction

Acromegaly is caused by excess growth hormone (GH) secreted from the pituitary gland most often due to a somatotropinoma. While somatotropinomas are monoclonal tumors that occur sporadically in the vast majority of the patients, acromegaly can also be part of a familial disease. No exact data are available in what percentage of unselected acromegaly patients have a congenital (germline or mosaic) cause of their disease. We know, however, that almost 50% of childhood-onset cases leading to gigantism have a now identifiable genetic background [1, 2].

Tumor development is a complex multistep process influenced by genetic, epigenetic and environmental factors as well as by the tumor microenvironment. In this brief overview, we concentrate on the genetic basis of sporadic and familial acromegaly while refer to an excellent review regarding other molecular mechanisms [3].

Acromegaly can be part of a syndromic disease occurring concomitantly with other endocrine tumors, such as in MEN1, MEN4, Carney complex, McCune-Albright and SDHx-related pituitary adenomas (Table 1) or presents as part of familiar isolated pituitary adenoma (FIPA) in aryl hydrocarbon receptor interacting protein (*AIP*) or *GPR101* (G-protein coupled receptor 101) mutation positive and negative cases [4, 5].

**Table 1:** Mutated genes associated with acromegaly/gigantism

Gene or genetic alteration	Gene location	Prevalence in pituitary tumors (%)	Prevalence in acromegaly (%)	Phenotype	Pathology
*AIP*	11q13.3	3.6%	50% in homogeneous FIPA 4% in apparently sporadic acromegaly	Familial isolated pituitary adenoma Younger patients; invasive tumors; worse response to SSA	Somatotropinoma
*MEN1*	11q13.1	0.6–2.6%	1.2%	Hyperparathyroidism, pituitary adenomas and pancreatic neuroendocrine tumors (multiple endocrine neoplasia type 1)	Somatotropinoma
*CDKN1B*	12p13.1	Rare	Rare	Hyperparathyroidism, pituitary adenomas and pancreatic neuroendocrine tumors (multiple endocrine neoplasia type 4)	Somatotropinoma
*PRKAR1A*	17q22-24	Only in acromegaly	65% of Carney complex patients	Acromegaly, cardiac and cutaneous myxomas, primary pigmented nodular adrenocortical disease and pigmented lesions of the skin and mucosae (Carney complex)	Somatotropinoma or hyperplasia
*SDHx*	SDHA 5p15.33 SDHB 1p36.13 SDHC 1q23.3 SDHD 11q23.1	Rare	Rare	Acromegaly and PGLs/phaeochromocytoma (3 PAs syndrome)	Somatotropinoma
GPR101	Xq26.3	1.6%	0–4.4%	X-linked acrogigantism: very early-onset gigantism	Somatotropinoma or hyperplasia
GNAS	20q13.3	Only in acromegaly	40%	Sporadic acromegaly and McCune-Albright syndrome	Somatotropinoma or hyperplasia

*AIP* aryl hydrocarbon receptor interacting protein, *MEN1* multiple endocrine neoplasia type 1, *CDKN1B* cyclin-dependent kinase inhibitor 1B, *PRKAR1A* protein kinase A regulatory subunit type I alpha, *PGLs* paragangliomas, *SDHx* genes of the succinate dehidrogenase family (A, B, C or D), *SSA* somatostatin analogs

### Familial isolated pituitary adenomas

Familial isolated pituitary adenoma syndrome is the most common familial cause of acromegaly/gigantism, as available data suggest that syndromic familial acromegaly, due to Carney complex, MEN1, MEN4 and SDH-related syndromes, are less common [6–10].

FIPA families can be heterogeneous (when more than one type of pituitary adenoma is present in the same family) or homogeneous [11]. In our cohort of 216 FIPA families, 60% had at least one subject with GH excess (92% of the 37 *AIP* mutation positive families and 53% of the 179 *AIP* mutation negative families). Twenty-eight percent of the whole cohort had homogenous GH FIPA (43% of the 37 *AIP* mutation positive families and 24% of the 179 *AIP* negative families).

**Fig. 1:**
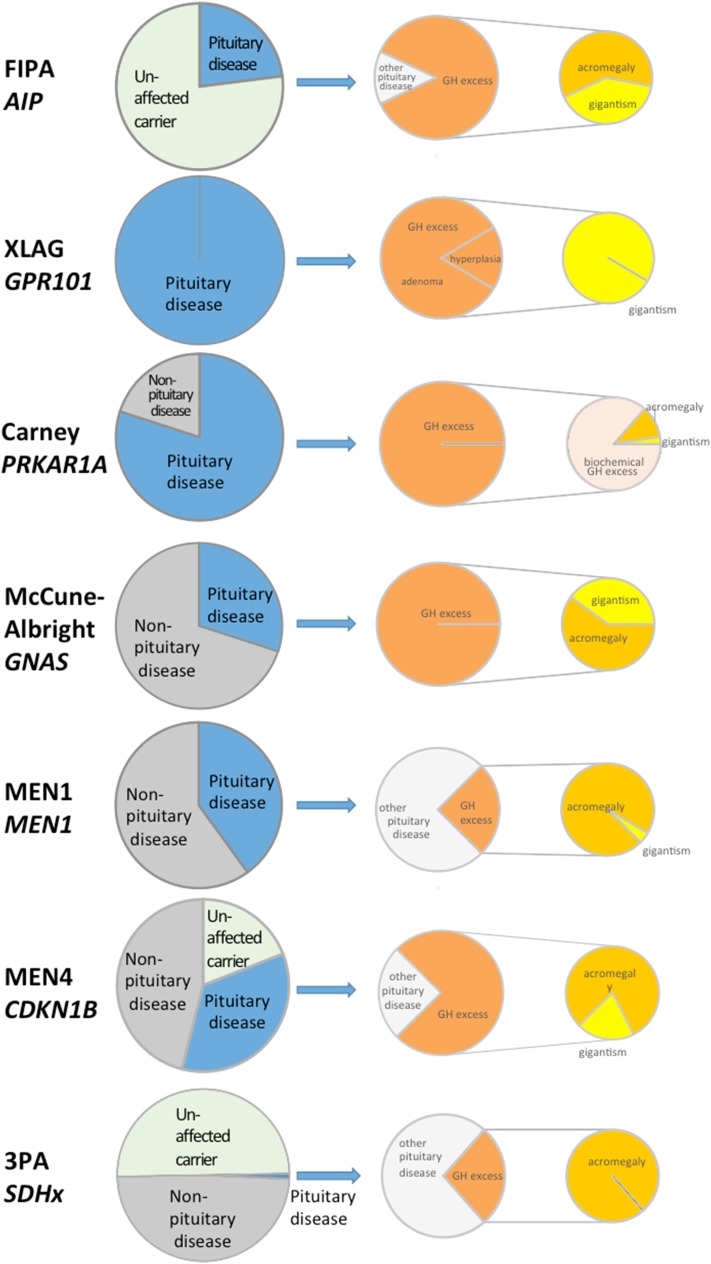
For each of the syndromes, the *left pie* represents the percentage of pituitary adenomas in patients with the disease. The *middle pie* represents the percentage of patients with GH excess and the *right pie* shows the proportion of patients with acromegaly or gigantism. AIP: 20 to 23% of gene carriers manifest the disease, all affected patients have a pituitary adenoma and 86% of these result in GH excess [6]. Overall 48% of GH excess patients have gigantism [6], but in a homogenous R304* cohort 58% had gigantism [14] confirming that truncating mutation patients have lower age of onset of disease [6]. XLAG: All gene carriers develop pituitary disease, 83% adenoma and 17% hyperplasia and all have gigantism [15]. Carney complex: 80% of gene carriers will have biochemical GH abnormality and 10% will manifest clinical acromegaly [16]. A few patient have been described with gigantism. McCune-Albright syndrome: 70% of gene carriers will have GH excess and 36% of these would have gigantism [17]. MEN1: 40% of MEN1 gene mutation carriers have a pituitary adenoma and 25% of these have acromegaly, with very few cases described in children [18, 19]. We note, however, that in a systematic screening study [10] a large number of small NFPAs have been identified and the percentage of acromegaly is only 7% among the pituitary adenoma patients in this study. GHRH-secreting pancreas tumors can also lead to acomegaly or gigantism in MEN1 patients. MEN4: Among the very few patients and unaffected family members described with *CDKN1B* mutation 4/5th of the patients were affected (this could be ascertainment bias and more data is needed to identify true penetrance), 63% of the affected subjects (30% of all carriers) had pituitary disease and 2/3 of these (5 cases) had GH excess with one of these having gigantism. Interestingly, in two of the five GH excess cases, the mutation was located in the 5′UTR (untranslated region) [20, 21]. 3PAs: As the penetrance of an abnormality in the various *SDH* subtype mutation carriers can be different, we show here penetrance data ~50% for *SDHB* mutations. Among the affected subjects very low percentage of *SDHx* carriers have a pituitary adenoma (<1%). Out of 15 cases reviewed four (28%) had acromegaly, none of them were childhood-onset [22]

The genetic basis for the majority of FIPA cases is currently not known. A fifth of the families have germline mutations in the *AIP* gene [6, 12] and *GPR101* mutations (duplications) have been described in two families [13] (Fig. 1).

### AIP

Heterozygous loss-of-function *AIP* mutations predispose the appearance of young-onset pituitary adenomas with an incomplete (20–23%) penetrance [23]. About 50% of *AIP* mutations kindreds present as sporadic cases due to low penetrance rather than *de novo* mutations [6]. The prevalence of *AIP* mutations is doubled (36–40%) when considering homogeneous acromegaly/gigantism FIPA families compared to the frequency in all the FIPA families. Somatotropinomas, with or without prolactin co-secretion, represent the most common *AIP* mutation positive adenoma type followed by prolactinomas (Fig. 1). A few clinically non-functioning adenomas have also been described but these usually show positive immunostaining for GH and prolactin [6]. A few ACTH- and a single TSH-secreting adenoma have also been described [24]. Somatotropinomas harboring *AIP* mutations are more frequently macroadenomas (88%) with extrasellar extension, more frequent in younger patients (78% younger than <30 years) and present with a higher frequency of apoplexy especially in children [6, 12, 25, 26]. A slight male preponderance has been shown earlier [12], but with extensive familial screening a more balanced picture is emerging [6]. Patients with *AIP* mutation show poor response to somatostatin receptor ligands (SRLs) [12]. There are two hypothesis to explain this poor response: (i) first-generation SRLs increase AIP expression and, in turn, this can upregulate the tumor suppressor zinc-finger protein ZAC1 which has been previously linked to SRLs anti-proliferative effects [27]. Interestingly, sporadic somatotropinomas without *AIP* mutations with low AIP protein expression also have a poor response to first-generation SRLs [28, 29]. Therefore, the lack or low level of AIP via the lack of ZAC1 upregulation could explain the resistance to treatment with SRLs [4, 30]; (ii) another mechanism for SRLs resistance of *AIP* mutation positive cases could be the low level of inhibitory G protein which has been identified both in embryonic fibroblast lacking *AIP* and human pituitary adenomas with *AIP* mutation [31].

The *AIP* gene is located at chromosome 11q13 and encodes a 330 amino acid protein suggested to be a tumor suppressor gene. AIP influences the cAMP pathway [32] via inhibitory G proteins (Gαi2) [31], via phosphodiesterases [33, 34] and via interfering with downstream pathway of somatostatin receptors [30], but the exact mechanism how lack of AIP leads to somatotroph adenomas is unclear. Over 70% of pathogenic mutations identified to date lead to missing or truncated protein (frameshift, nonsense, small and large deletions, variants in the regulatory region with functional data on promoter activation supporting reduced activity), while missense mutations and in-frame small deletions and insertions can also occur mostly affecting the C terminal characteristic tetratricopeptide domains [4, 24, 25]. Separating pathogenic variants from benign polymorphisms can be challenging in some cases. Several *AIP* sequence variants were tested in vitro using two-hybrid assay between PDE4A5 and AIP to help to identify pathogenicity of some of the questionable *AIP* variants [25, 33]. There are a few mutational hotspots in the *AIP* gene: the most common one affects the Arg304 residue, while Arg81 and Arg271 have also been described in independent cohorts [35].

The type of *AIP* mutation can influence the phenotype, with patients harboring truncating mutations being younger at diagnosis than those harboring full-length mutated proteins [6]. Regarding mutant AIP proteins, it has been shown that many pathogenic missense *AIP* mutations have a significantly reduced stability in experimental conditions [36]. This suggests that the mechanism of lack of function in these cases may be the rapid degradation of the (probably) misfolded protein [36].

AIP mutations can also be identified in sporadic acromegaly cases [6, 37–41]. Considering patients without any age limitations, only about 4% of the apparently sporadic patients will harbor a germline *AIP* mutation [40]. However, this is more frequent in young patients, especially those harboring macroadenomas, being found in up to 13% of patients younger than 30 years of age and can be found in up to 33% of pediatric (18 years) patients [39, 42]. The lack of family history is due to the incomplete penetrance as only one de novo acromegaly case has been described to date [43].

In the presence of an *AIP* mutation in a patient with apparently sporadic acromegaly, screening of the first-degree relatives for the mutation should be performed [44]. Family member screening of *AIP* carriers was shown to identify 24% of ‘unaffected’ carriers with biochemical or radiological abnormalities, and, interestingly, half of these actually had symptoms but did not previously seek medical attention. We need to be cautious, however, as, similar to MEN1 screening, the small non-functioning lesions in *AIP* mutation carrier subjects may represent incidentalomas. Considering the prevalence of germline *AIP* mutations in young patients with apparently sporadic pituitary tumors, while no formal guidelines exist, several groups recommend that screening for *AIP* mutations be performed in all patients diagnosed before 18 years of age and in patients harboring a macroadenoma diagnosed before 30 years of age [24, 40, 44].

### X-linked acrogigantism (XLAG)

Patients with very early-onset gigantism may harbor an unusual type of mutation, a microduplication of chromosome Xq26.3 (Fig. 1) [13]. The Xq26.3 region codes four genes, but only one of them, *GPR101*, coding for an orphan G-protein coupled receptor, was overexpressed in the patients with a microduplication of this region [13]. The causative role of this gene was reinforced by a case with typical phenotype but with a smaller microduplication only including the *GPR101* gene [2]. Extensive search for Xq26.3 microduplication in pediatric or adult cases of GH excess could only identify this genetic abnormality in very early-onset (accelerated growth before the age of 5 years) gigantism cases. Altogether 31 patients have been reported to date, including two unrelated families and 26 sporadic cases [2, 13, 45–47]. The majority of the cases are females and harbor de novo mutations and penetrance is 100% based on available data. Sporadic male cases described to date harbor mosaic mutations [2, 45, 48]. Interestingly, diagnosis was challenging in a case of a 4 year old boy with pituitary hyperplasia and gigantism, where germline DNA showed no detectable duplication with CGH array and droplet PCR was not conclusive. The diagnosis was finally made using DNA derived from pituitary tissue and skin biopsy showing the typical *GPR101* duplication [48]. Patients have a median age of disease onset of 1.0 year (ranging from 0.5 to 2.0 years), and were younger than those gigantism patients who did not present the microduplication (median age of disease onset of 16.0 years, ranging from 5.0 to 18.0 years) [1, 13]. XLAG patients represent the second largest group of patients with childhood-onset acromegaly, after *AIP* mutation positive patients and there is no phenotypic difference between mosaic or germline XLAG cases [1, 2, 45].

The *GPR101* gene variant p.E308D was originally suggested to play a role in acromegaly pathogenesis (11 out of 248 patients with acromegaly) (4.4%) [13], but extensive data from different laboratories cannot confirm these data. A large study including 766 patients with apparently sporadic pituitary tumors found a frequency of 1.6% of germline *GPR101* variants (three patients with acromegaly, two with Cushing`s disease and one with a nonfunctioning pituitary adenomas) [49, 50]. The observed allele frequency (0.49) was not different form the frequency present in the general population [0.69, Exome Aggregation Consortium (ExAc) database (http://exac.broadinstitute.org)] in another study which included 395 leukocyte- and 193 somatotropinoma-derived samples [2]. A recent Italian study found no germline *GPR101* variants in a cohort of 215 patients with GH-secreting adenomas [38].

### Multiple endocrine neoplasia types 1 and 4 (MEN1 and MEN4)

Acromegaly can occur in the setting of a multiple endocrine neoplasia. In multiple endocrine neoplasia type 1 (MEN1), pituitary adenomas are associated with hyperparathyroidism and neuroendocrine tumors of the pancreas [19]. Germline mutations in the *MEN1* gene are found in 80–90% of the probands with familial MEN1 and in approximately 65% of the patients with sporadic MEN1 [19, 51, 52]. *MEN1* mutations were described in 1.2% of sporadic acromegaly patients younger than 30 years of age [51].

Primary hyperparathyroidism is the most common component of MEN1, being observed in over 90% of the cases. Pituitary tumors occur in 38% (15–50%) of the cases presenting with pituitary disease [10, 53, 54], with prolactinomas being the most frequent (60%) and somatotropinomas being the second most common (25%) (Fig. 1) [19]. Interestingly, a prospective screening study found different proportions of pituitary disease subtypes with significantly higher number non-functioning adenomas. These differences might be explained by the different strategies used to collect the data (spontaneous presentation vs. clinical screening) [10] and it is unclear what percentage of these often small non-functioning adenomas represent, in fact, incidentalomas. Pituitary tumors in MEN1 occur more frequently in women, prolactinomas are more common in younger patients and are often invasive macroadenomas. In MEN1 patients with acromegaly, the diagnosis is usually made after 40 years of age [19]. Although three patients with MEN1-associated acromegaly are reported to be treated medically, no data are available of their SRLs responsiveness [8]. In MEN1 patients with acromegaly and enlarged pituitary gland with no discernable pituitary adenoma, a GHRH-secreting neuroendocrine tumor, typically arising from the pancreas should also be considered [55]. Co-existance of acromegaly due to GHRH-secreting neuroendocrine tumor and a prolactinoma has also been described in MEN1 syndrome, representing a significant diagnostic challange [56]. Circulating GHRH measurement is now routinely available in reference laboratories [55].

The tumor suppressor gene *MEN1* is located at chromosome 11q13 coding for the protein menin. MEN1 exhibits an autosomal dominant inheritance pattern [19, 57]. To date, the exact mechanism by which inactivating *MEN1* mutation leads to tumorigenesis is unknown, but menin has been shown to influence numerous relevant processes such as the cell cycle, cell division, genome stability and transcriptional regulation [57]. The last detailed review identified 1133 different germline and 203 somatic mutations without typical hotspots [51], although codons 139, in exon 2, and 418 in exon 9, were affected by the highest number of mutations (seven different mutations) [51]. Regarding the type of mutations, 26% were missense mutations, 14% were nonsense mutations, 42% were frameshift insertions or deletions, 5.5% were in-frame insertions or deletions, 10% were splice site mutations and 2.5% were large deletions [51, 52]. Screening for *MEN1* mutations in patients with acromegaly should be considered in the presence of other features of the syndrome or in the presence of a family history of MEN1 [57, 58].

Rarely patients with MEN1 phenotype, but without *MEN1* mutations, harbor mutations in the cyclin-dependent kinase inhibitor 1B (*CDKN1B*) gene coding for p27^kip1^ protein [9, 59]. This syndrome is named multiple endocrine neoplasia type 4 (MEN4) and only nine cases have been described with pituitary adenomas. Five of the nine reported MEN4-related pituitary disease is acromegaly (Fig. 1). Therefore, in an acromegaly/gigantism patient with features of the MEN1 syndrome but without *MEN1* mutations, screening for *CDKN1B* mutations is indicated [58].

### Carney complex

Carney complex (CNC) is a syndrome characterized by the presence of acromegaly (10–12%), cardiac and cutaneous myxomas, primary pigmented nodular adrenocortical disease (PPNAD) and pigmented lesions of the skin and mucosae (can vary from lentigines to blue nevi) [16]. CNC presents as familial disease in 70% of the cases and is inherited in an autosomal dominant manner with an overall full penetrance. GH excess is present in 80% of the cases and is mainly consequence of a pituitary somatotroph hyperplasia and not due to a pituitary adenoma (Fig. 1) [16].

The cause of the majority of CNC cases is a mutation in the *PRKAR1A* gene, coding for the regulatory subunit type I alpha of the protein kinase A, located at chromosome 17q24 [60, 61]. A second genetic locus associated with CNC has been described in chromosome region 2p16, but the specific genetic defect has not been elucidated [16, 61]. A single case of gene duplication affecting the catalytic B subunit of PKA has also been described [62].

### Acromegaly and paraganglioma/phaeochromocytoma syndrome

The coexistence of acromegaly with paragangliomas (PGLs) or phaeochromocytomas has been described since 1952 [63]. As they are both rare tumors, the occurrence of both in the same patient can be coincidental but attention in the recent years has been given for a possibly common genetic basis, for the recently named “3PAs” syndrome (paragangliomas, phaeochromocytomas and pituitary adenomas) [64].

Considering that mutations in the genes coding the succinate dehydrogenase (SDH) complex are the most common genetic causes of PGLs/phaeochromocytomas it has been studied in the cases of 3PAs [22, 64, 65]. Following description of a succinate dehydrogenase type B (*SDHB)* mutation positive patient with a pituitary adenoma [66] and *SDHB* mutation positive familial PGLs and macroprolactinomas [67], somatotroph adenomas have also been associated with *SDH* mutations (*SDHA, SDHB and SDHD)* [64, 65, 68, 69].

In some cases, the 3PAs syndrome occurs in the absence of mutations in the SDH complex genes [22]. Mutations in genes causing other familial syndromes may be found and, actually, there is a reported case with mutation in the *MEN1* and other case with mutation in the *RET* gene [65, 70].

### McCune-Albright syndrome

Patients with McCune-Albright syndrome may present as sporadic acromegaly or gigantism. They harbor a mosaic mutation in the *GNAS* gene coding for the alpha-subunit of the G-stimulatory protein (Gαs). McCune-Albright syndrome characterized by the development of polyostotic fibrous dysplasia, *café-au-lait* spots and endocrine hyperfunction, including precocious puberty, thyrotoxicosis due to autonomous thyroid nodules, Cushing`s syndrome (caused by nodular adrenal hyperplasia) and GH excess or manifest acromegaly/gigantism [71, 72]. Excess of GH, often with prolactin co-secretion, is observed in approximately 20% of the cases. In the majority of the cases pituitary hyperfunction is caused by pituitary hyperplasia and not by an adenoma [72]. The diagnosis of McCune-Albright syndrome, thus, should be suspected in acromegaly/gigantism patients with other features of the syndrome.

The *GNAS* gene is located at chromosome 20q13.3. Mutations in the *GNAS* gene have been described more frequently at codon 201 (Arg201 for Cys or His or Ser) than at codon 227 (Gln227 for Arg or Leu) [73–75]. These mutations lead to loss of GTPase activity of the G-stimulatory protein alpha subunit while leaving the adenyl cyclase stimulatory activity intact. The resulting constitutive activation and increased intracellular cAMP levels lead to somatotroph proliferation/hyperplasia and GH hypersecretion [76].

### GH excess in neurofibromatosis type 1

GH excess has been described in both pediatric and adult patients with neurofibromatosis leading to gigantism or acromegaly ([77–80] and references within). Although most cases are associated with optic gliomas and no pituitary tumors, a few has been described with GH positive pituitary adenomas and it is unclear if the latter ones are pure coincidences or not.

## Somatic changes in somatotropinomas

The most common somatic mutation in acromegaly are the activating mutations of the *GNAS* gene [76], with a frequency of about 40% (ranging from 10 to 50% depending on the ethnical background) [73, 74, 81–83]. Somatic *GNAS* mutations are found with equal frequency in both sexes and are more frequent in tumors from older patients [73, 83]. They are also more common in smaller tumors that secrete higher GH levels and are associated with a densely granulated pattern in the histopathology analysis [75, 84].

Some studies suggest that tumors harboring a *GNAS* mutation present a better response to first-generation SRLs, but this finding is not homogeneous in the literature [74, 83–85]. Recently, a meta-analysis including eight studies and 315 patients concluded that patients with a *GNAS* mutation present a higher reduction of GH levels during the acute octreotide suppression test [75]. However, studies addressing the response to long-term SRLs treatment present conflicting results [74, 84].

To identify further somatic mutations in sporadic somatotroph adenomas exome [83, 86] and whole genome sequencing studies were performed [87]. Välimäki et al. performed a whole-genome sequencing and single-nucleotide polymorphism (SNP) array in 12 fresh-frozen somatotropinomas and in the leucocyte DNA of the corresponding patients [87]. The whole-genome sequencing showed an average of 129 somatic nucleotide variants (SNV), with an average of 2.3 SNV per tumor. However, the only recurrent somatic events were *GNAS* mutations and shared chromosome losses (chromosomes 1, 6, 13, 14, 15, 16, 18 and 22). No novel recurrent mutated genes were identified. In agreement with these findings, Ronchi et al. performed a next-generation whole-exome sequencing in 36 sporadic somatotropinomas, identifying a median of three mutations per sample [86]. Again, the only recurrent somatic mutation observed was in the *GNAS* gene (31.4% of the cases). More recently, Song et al. performed whole-exome sequencing and copy number analysis in 125 pituitary adenomas, including 20 somatotropinomas [83]. They observed *GNAS* mutations in 55% of the tumors. Interestingly, copy number variation was also found affecting the chromosome 20q13.3 region, where the *GNAS* gene is located, in 8/20 samples, 5 of those also had a single nucleotide *GNAS* mutation. However, the 20q13.33 locus contains synaptonemal complex protein 2 (*SYCP2*), a cohesion complex gene, while other cohesion complex genes were also found amplified in somatotroph adenomas:*SYPC1* on chromosome 1p13.2 was amplified in 11/20 somatotroph samples and RAD21 cohesin complex component like 1 (*RAD21L1*) on chromosome 20p13 was amplified in 4/20. While data from independent cohort and functional characterization is needed, these results raise the possibility that cohesion complex might play a role in pituitary adenoma genesis [88].

These studies altogether identified three possible pathways involved in somatotroph tumorigenesis: the cAMP, the calcium-channel signaling and the cohesin pathways. In the study by Ronchi et al., seven genes involved in the cAMP signaling pathway were affected in 14 of the 36 samples and eight samples harbored variants in genes involved in the calcium signaling or metabolism [86]. In the study by Välimäki et al., five genes involved in cAMP or calcium-related signaling pathways were found to be mutated [87]. As an increase in cAMP levels is associated with an increase in cytosolic free calcium and this triggers the GH secretion, it is probably that genetic and/or epigenetic alterations in these pathways lead to the development of somatotropinomas. In the Song et al. study 13/20 samples had changes in the cohesion pathway members [83].

## Conclusion

Acromegaly is caused in the majority of the cases by a sporadic somatotropinoma and rarely by pituitary hyperplasia. In 95% of the cases it occurs sporadically but almost 50% of the childhood-onset cases have an identifiable genetic background, most commonly *AIP* or *GPR101* mutations. Acromegaly is one of the most frequent pituitary adenoma types which occur in a familial setting most commonly due to *AIP* mutations. Genetic screening has been shown to identify family members in an earlier stage of the disease which is predicted to lead to better long-term outcome. Therefore genetic testing and counselling of family members will improve the long-term management of this disease.
